# Manipulation of the Alternative NF‐κB Pathway in Mice Has Sexually Dimorphic Effects on Bone

**DOI:** 10.1002/jbm4.10066

**Published:** 2018-08-23

**Authors:** Allahdad Zarei, Chang Yang, Jesse Gibbs, Jennifer L Davis, Anna Ballard, Rong Zeng, Linda Cox, Deborah J Veis

**Affiliations:** ^1^ Musculoskeletal Research Center Division of Bone and Mineral Diseases Department of Medicine Washington University School of Medicine St. Louis MO USA; ^2^ Department of Pathology and Immunology Washington University School of Medicine St. Louis MO USA

**Keywords:** ALTERNATIVE NF‐κB PATHWAY, NIK, RelB, IAP ANTAGONIST, SEXUAL DIMORPHISM

## Abstract

Alternative NF‐κB signaling promotes osteoclastogenesis and pathological bone loss, but the effect of sex on phenotype has not been explored. We disrupted alternative NF‐κB signaling by deletion of upstream kinase NF‐κB‐inducing kinase (NIK) or NF‐κB subunit RelB and found that both NIK‐deficient and RelB‐deficient female mice possessed more than twofold higher trabecular bone mass compared to controls, whereas no differences were observed in males. In vitro, RelB‐deficient precursors from female mice showed a more severe osteoclast (OC) differentiation defect than male, while WT had no sex bias. Next, we asked whether pharmacologic activation of alternative NF‐κB by inhibitor of apoptosis (IAP) antagonist BV6 has sex‐dependent effects on bone. Unlike male mice that lost bone, female mice on BV6 for 4 weeks showed no changes in either trabecular bone mass or OC number. Because estrogen generally suppresses NF‐κB, we hypothesized that estrogen protects bone from BV6 effects in vivo. Thus, we performed ovariectomy or sham surgery in female mice, then treated with BV6 or vehicle for 4 weeks. Although ovariectomy caused bone loss, BV6 did not have any additional impact, suggesting that direct estrogen effects do not cause resistance to BV6 in vivo. The osteopenic effects of IAP antagonists in males may have implications for their use in cancer therapy. © 2018 The Authors. *JBMR Plus* published by Wiley Periodicals, Inc. on behalf of American Society for Bone and Mineral Research.

## Introduction

Alternative NF‐κB is an important regulator of osteoclastogenesis.^1^ The apex kinase in this pathway is NF‐κB–inducing kinase (NIK), which activates IκB kinase α (IKKα) resulting in the nuclear translocation of RelB/p52 dimers that contribute to osteoclast (OC) differentiation. NIK is negatively regulated by cellular inhibitor of apoptosis proteins (cIAPs), which target it for degradation by the proteasome.^2^ Various human cancers have been shown to express high levels of cIAPs and alteration in cIAPs is associated with tumorigenesis and resistance to chemotherapy.^3^ IAP antagonists, also known as second mitochondrial‐derived activator of caspases (SMAC) mimetics, are now under development as anticancer drugs,^4^, ^5^ making it valuable to understand their effects in vivo. A major complication for patients with malignant tumors such as lung, breast, and prostate cancer is bone metastasis.^6^ Crosstalk between cancer cells and bone cells provides a favorable environment for tumor growth. Osteoclasts are stimulated by tumor‐produced cytokines that also stimulate receptor activator of NF‐κB ligand (RANKL) production by osteoblasts.^7^ We previously found that the IAP antagonist family of drugs activates NF‐κB in osteoclasts, enhances osteoclastogenesis, and promotes bone metastasis in male mouse models.^8^ We have also shown that tumor‐free male BALB/c and C57BL/6 mice receiving BV6, an IAP antagonist, over 4 weeks develop high turnover osteoporosis, dependent on the presence of NIK.^8^ Thus, IAP antagonists seem to provide a more favorable microenvironment for tumor growth in bone.

Fundamental differences in the musculoskeletal system exist between males and females, becoming apparent primarily at the time of sexual maturity, when males develop higher skeletal size, mass, and strength.^9^ The sexual dimorphism in bone has been attributed mainly to direct actions of sex hormones, and bone cells express receptors for sex hormones as well as sex hormone–metabolizing enzymes.^10^ Sex hormones may also act indirectly by interacting with effectors such as insulin‐like growth factor, inflammation, oxidative stress, or the central nervous system.^10^

Previous studies of mice with alterations in the alternative NF‐κB pathway, including our own, did not consider sex as a variable, using either all male or mixed sex cohorts.^8^, ^11^, ^12^, ^13^, ^14^ Global loss of NIK or RelB activity led to an increase in trabecular bone mass, whereas stimulation of the pathway led to bone loss, both primarily attributed to changes in OC formation and/or function. Work in other fields has shown important effects of estrogen on NF‐κB signaling,^15^, ^16^ including interactions with RelB‐mediated events.^17^ Because OCs express functional estrogen receptors (ERαs),^18^ we hypothesized that the role of alternative NF‐κB in bone would be impacted by estrogen status. In the current study, we investigate whether modulation of alternative NF‐κB genetically or pharmacologically (with an IAP antagonist) has any sexual dimorphic effects on bone in mice.

## Materials and Methods

### Reagents and mice

All chemical and reagents were purchased from MilliporeSigma (St. Louis, MO, USA) unless otherwise mentioned. BV6 was synthesiaed at the Department of Biochemistry and Molecular Biophysics, Washington University. CMG 14‐12^19^ cell supernatant was used as the source of M‐CSF and RANKL was made as described.^20^ NIK‐knockout (KO),^21^ RelB‐KO,^22^ BALB/c, and C57BL/6 (Jackson Laboratories) mice were maintained in a specific pathogen‐free facility as described.^14^ All in vivo experimental protocols were carried out in accordance with the relevant guidelines and regulations and approved by the Institutional Animal Care and Use Committee at Washington University School of Medicine.

### Cell culture and BV6 treatment

Mouse bone marrow macrophages (BMMs) were expanded from whole bone marrow in a 1:10 dilution of CMG 14‐12 cell supernatant (source of M‐CSF) and then plated at 1 × 10^4^/ well in 96‐well‐plate formats in a 1:50 dilution of CMG 14‐12 cell supernatant and 10 ng/mL RANKL with media replaced every day. TGFβ (1 ng/mL) and TNFα (10 ng/mL), both from R&D Systems, Minneapolis, MN, USA, replaced daily in addition to MCSF and RANKL, were used to potentiate osteoclastogenesis in vitro.^23^, ^24^, ^25^ BV6 (8 μM) or vehicle was added into the OC culture media and incubated with cells for 2 hours every day, then cell culture was replaced with OC culture media (MCSF + RANKL) without BV6. This procedure was repeated for 4 days when cell cultures were stopped, fixed with 4% paraformaldehyde + 0.1% Triton X‐100 in PBS for 10 min and stained for tartrate‐resistant acid phosphatase (TRAP) activity using a kit (Sigma) following the manufacturer's instructions. Plates were photographed with a OLYMPUS U‐CMAD3 camera and TRAP‐positive multinucleated cells with more than three nuclei were counted as OCs with a OLYMPUS U‐CMAD3 camera.

To assess the extent of resorption, BMMs were cultured at 7 × 10^3^ in 96‐well plates on bone slices. Cells were maintained in a 1:50 dilution of CMG 14‐12 cell supernatant and 15 ng/mL RANKL ± 1 ng/mL TGFβ for 5 days. Bone slices were washed with PBS and fixed with 4% paraformaldehyde for 10 min, and cells were removed with 30 s incubation with 2N NaOH. Resorption pits were stained with peroxidase‐conjugated wheat germ agglutinin (Sigma) at 37°C for 30 min and developed with DAB (3,3′‐diaminobenzidine) solution at 37°C for 30 min. Bone slices were washed with PBS, air dried, and photographed with a OLYMPUS U‐CMAD3 camera.

### Real‐time RT‐PCR

Total RNA of BMMs grown at 2 × 10^5^ per well for 24 hours in M‐CSF or 3 days in MCSF +RANKL ± TGFβ was extracted with NucleoSpin RNA II kit (Machery‐Nagel, Bethlehem, PA, USA), and cDNA synthesis was performed with RNA to cDNA EcoDry™ Premix (Takara Bio, Mountain View, CA, USA). RT‐PCR was performed on C1000™ Thermal Cycler (Bio‐Rad, Hercules, CA, USA) using iTaq Universal SYBER Green Supermix (Bio‐Rad) for 40 cycles with denaturation at 95°C for 5 s, and annealing/extension at 60°C for 31 s using the following:

TRAP 5′‐CGTCTCTGCACAGATTGCAT‐3′, 5′AAGCGCAAACGGTAGTAAGG‐3′; MMP9 5′‐ACGACATAGACGGCATCCA‐3′, 5′‐GCTGTGGTTCAGTTGTGGTG‐3′; cathepsin K (Ctsk) 5′‐ATGTGGGTGTTCAAGTTTCTGC‐3′, 5′‐CCACAAGATTCTGGGGACTC‐3′ primers. The threshold cycle (Ct) values were normalized to the housekeeping genes B2M (5′‐CTGCTACGTAACACAGTTCCACCC‐3′, 5′‐CATGATGCTTGATCACATGTCTCG‐3′); and HMBS (5′‐GAGTCTAGATGGCTCAGATAGCATGC‐3′, 5′‐CCTACAGACCAGTTAGCGCACATC‐3′) and the relative expression was calculated using the delta‐delta Ct method.

### Ovariectomy

Forty 3‐month‐old female BALB/c mice weighing 21.5 ± 1.3 g were purchased from Jackson Laboratories. Mice were housed in a room with controlled temperature of 23°C and 12‐hour/12‐hour light‐dark cycle with access to clean water and standard pellet diet. After a week of acclimatization, all mice were subjected to ovariectomy (OVX) or sham surgery under sterile conditions. All mice were anesthetized under isoflurane gas and administered a single dose of 1 mg/kg Buprenex‐SR. Bilateral ovariectomy of 20 mice involved the removal of both left and right ovaries, whereas in sham groups (*n* = 20) surgery involved skin and peritoneal incision, leaving the ovaries intact.

After surgeries mice were allowed to recover for a week and randomly divided into four groups (sham‐vehicle, sham‐BV6, OVX‐vehicle, and OVX‐BV6), 10 mice in each group, housed in eight cages with each cage containing both sham and ovariectomized mice. Mice were either injected with BV6 (10 mg/kg) or vehicle intraperitoneally once a week over 4 weeks and body weights were recorded every week prior to injections to adjust dosage. Mice were euthanized 38 days after surgery. Right and left femurs and tibias were dissected for μCT and histomorphometric analysis.

### Histomorphometric analysis

Femurs and tibias were fixed in 10% formalin overnight, embedded in methyl methacrylate, and sectioned. Analysis was conducted by a blinded observer using BIOQUANT OSTEO 2017 software (BIOQUANT Image Analysis Corporation).

### μCT analysis

Tibias were fixed overnight in 10% formalin, washed with PBS, and stored in 70% ethanol at 4°C for μCT40 analysis. Tibias of BALBc mice treated with BV6 were scanned with in vivo μCT (vivaCT; Scanco Medical) and RelB (tibia), NIK (femur) wild‐type (WT)/heterozygous (HT)/knockout (KO) as well as sham and ovariectomized tibias by μCT40 at 55 kVp, 10 μm resolution. Lower threshold of 205 and upper threshold of 1000, Gauss sigma of 0.4, Gauss support of 1 for vivaCT and Gauss sigma of 0.4 and Gauss support of 1 for μCT40 were used. For μCT, regions of interest were selected immediately distal (tibia) or proximal (femur) to the growth plate extending 150 slices for RelB and NIK‐deficient mice and 100 slices for OVX mice. All vivaCT analyses were done with regions of interest immediately distal to proximal tibial growth plate extending 50 slices.

### Statistical analysis

Data are presented as mean ± SD with sample number indicated in each figure legend. Two‐way analysis of variance (ANOVA) followed by Sidak's multiple comparisons test was used for comparison between groups. Statistical analyses were carried out using GraphPad Prism 7 (GraphPad, La Jolla, CA, USA). A *p* value of less than 0.05 was considered significant.

## Results

### Genetic loss of alternative NF‐κB increases trabecular bone mass in female mice, but not males

**Figure 1:**
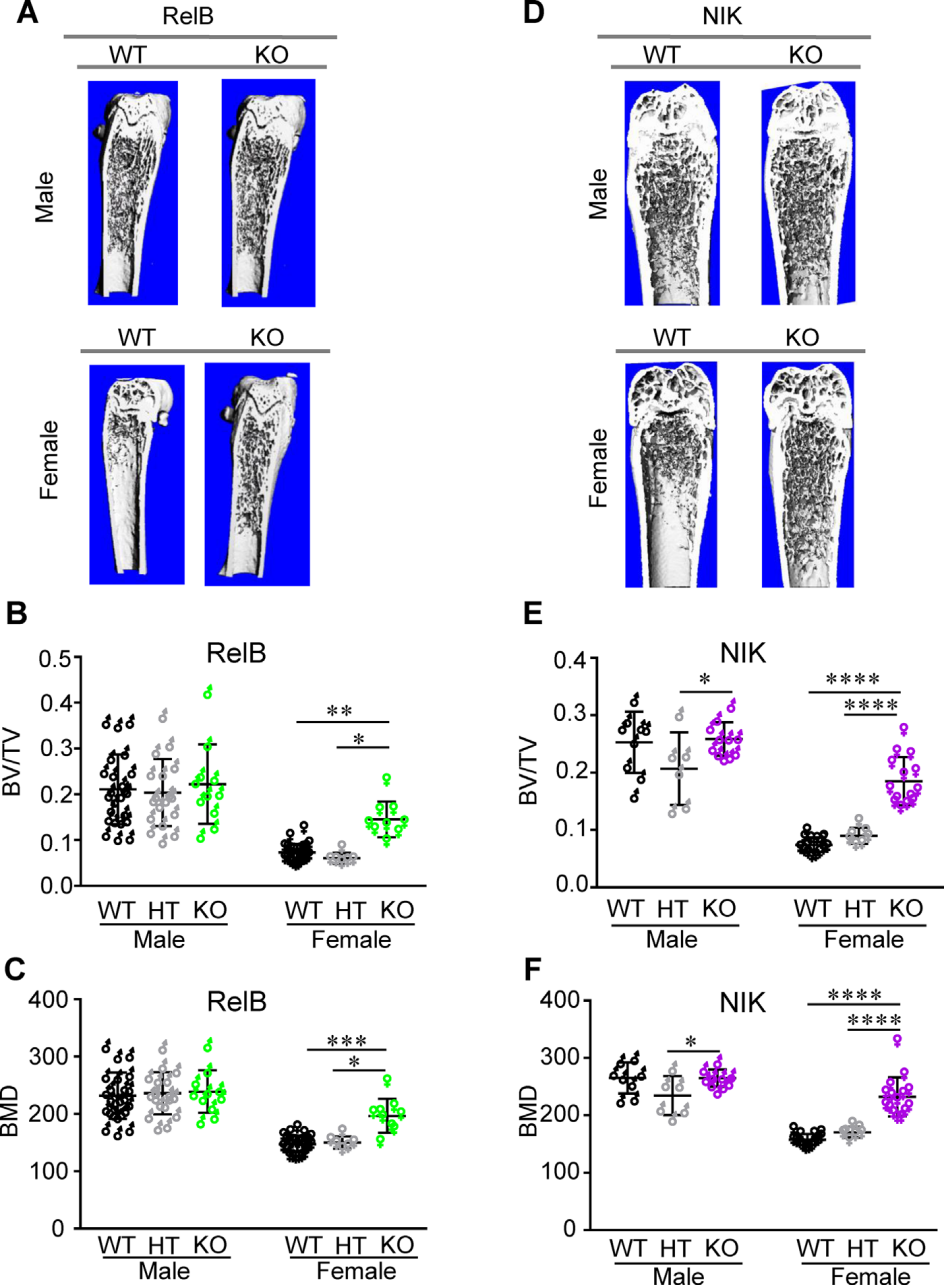
Increased trabecular bone volume and bone mineral density in RelB‐KO and NIK‐KO female mice. μCT was performed on tibiae of RelB WT, HT, and KO mice (*A*–*C*) and femurs of NIK‐WT, HT, and KO mice (*D*–*F*), all at 10 weeks of age. Representative three‐dimensional μCT reconstructions of median RelB WT and KO (*A*) and NIK WT and KO mice (*D*). Trabecular BV/TV (*B*,*E*) and BMD (*C*,*F*). Data was analyzed by two‐way ANOVA, with comparisons within each sex. *p < 0.05, **p < 0.01, ***p < 0.001, and ****p < 0.0001. BV/TV = bone volume/total volume; BMD = bone mineral density.

We first collected tibia samples in both sexes at 10 weeks of age from RelB‐deficient mice and performed μCT ex vivo (Fig. 1 *A*–*C*). There were no significant differences in any trabecular bone parameters between WT, HT, or KO male mice. However, female RelB‐KO mice had 2.3‐fold higher trabecular bone volume (BV/TV; *p* < 0.01) and 1.4‐fold higher bone mineral density (BMD; *p* < 0.001) compared to WT and HT littermates. A similar analysis of the femurs of NIK‐deficient mice was strikingly parallel to the data from the RelB‐deficient colony, with only female NIK‐KO mice showing an increase in trabecular bone parameters compared to WT controls (Fig. 1 *D*–*F*). Thus, there is strong sexual dimorphism in the response of bone to loss of alternative NF‐κB signaling.

### In vitro osteoclastogenesis is more suppressed in female RelB‐deficient and NIK‐deficient cultures than male

**Figure 2:**
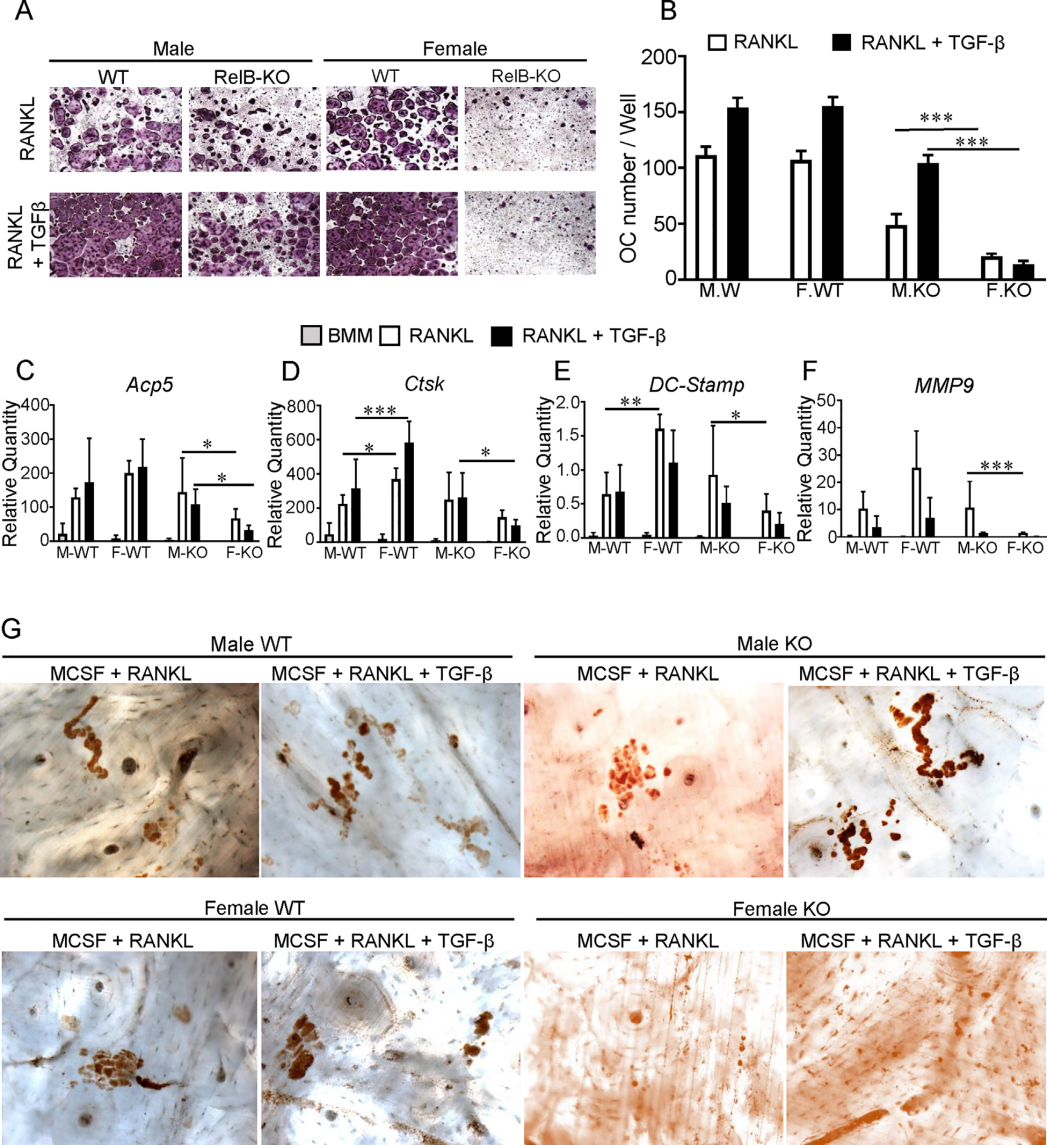
Increased severity of osteoclast defect in female RelB‐KO mice in vitro. (*A*) RANKL‐induced (top row) osteoclast formation from male and female RelB‐KO mice compared to WT littermates, demonstrate a more dramatic decrease in OCs in female RelB‐KO. Addition of TGF‐β with RANKL (bottom row) does not normalize the difference between male and female RelB‐KO. (*B*) TRAP positive cells with ≥3 nuclei in *A* were counted in each well and plotted, white indicates RANKL only, black RANKL + TGFβ. OC differentiation markers (*C*) Acp5 (Trap), (*D*) CatK, (*E*) DC‐Stamp, and (*F*) MMP9 were assessed at transcript levels from BMMs of 3 male WT (M‐WT), 3 male knockout (M‐KO), 3 female WT (F‐WT), and 3 female knockout (F‐KO) RelB mice after 3 days of culture and plotted against BMM as baseline. (*G*) Representative images of bone resorption from samples in *C*–*F* after 5 days of cell culture at magnification ×10. Data presented as mean ± SD, two‐way ANOVA, *p < 0.05, **p < 0.01 and ***p < 0.001. Significant differences for comparison between RANKL and RANKL + TGFβ data within each group is not shown, * for comparison between male and female.

To further investigate the phenotypes seen in vivo, OC precursors (bone marrow macrophages, BMMs) isolated from both male and female RelB‐KO mice were cultured in the presence of RANKL and M‐CSF. No significant differences between male and female OC formation were observed in BMMs isolated from WT mice (Fig. 2 *A*, top row, Fig. 2 *B*, white bars). OC formation was significantly hampered in response to RANKL in RelB‐KO BMMs isolated from both sexes, but the effect was more pronounced in female cultures. Addition of TGFβ to the osteoclastogenic cultures increased the number of OCs generated in all cases except for RelB‐KO females (Fig. 2 *A*, bottom row, Fig. 2 *B*, black bars).

Osteoclast differentiation markers were upregulated in cells from both male and female WT mice in the presence of M‐CSF, RANKL ± TGFβ (Fig. 2 *C*–*F*). Despite similar OC numbers, female WT cells expressed higher levels of Ctsk compared to male WT. Consistent with OC number, there was significantly less induction of all four osteoclast differentiation markers in cells isolated from female RelB‐KO mice compared to their male KO counterparts. Similar to low OC gene expression in female knockout cells, no resorption pits were detected from these female‐KO cultures grown either in the presence of MCSF+RANKL or M‐CSF+RANKL+TGFβ (Fig. 2 *G*).

BMMs isolated from either male or female NIK‐KO mice generated few OCs in the presence of RANKL and M‐CSF (Supplementary Fig.  1A), suggesting osteoclastogenesis is hampered in both sexes. However, similar to the results with RelB‐KO cultures, multinucleated TRAP‐positive cells were significantly increased in cultures of male, but not female, mice when media was further supplemented with TNFα (Supplementary Fig.  1B). Thus, the differential sensitivity of males and females to loss of alternative NF‐κB signaling is present both in vivo and in a cell autonomous fashion in vitro.

### Female mice are protected from the osteolytic effect of BV6 in vivo

**Figure 3:**
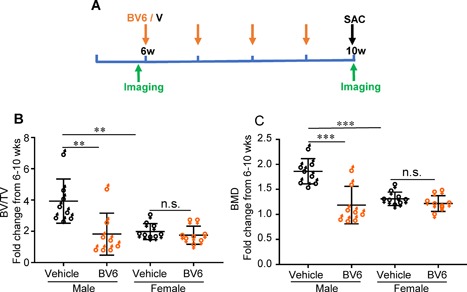
Only male mice treated with IAP antagonist BV6 lose bone. (*A*) WT BALB/c mice, age 6 weeks, male and female (*n* = 7–8/group) were given 4 weekly IP injections of BV6 (orange) (10 mg/kg) or vehicle (black). vivaCT was used to calculate fold changes from 6 to 10 weeks for each animal in (*B*) BV/TV and (*C*) BMD. Mean ± SD, two‐way ANOVA, **p < 0.01 and ***p < 0.001. n.s. = not significant.

In the current study, we explored the effects of BV6 versus vehicle in both male and female BALB/c mice treated from 6 to 10 weeks of age (Fig. 3 *A*). Consistent with our earlier experiments, pretreatment and posttreatment vivaCT analysis in males injected with BV6 over 4 weeks showed significantly lower BV/TV (*p* < 0.01) and BMD (*p* < 0.001) (Fig. 3 *B*, *C*) compared to male mice on vehicle. In contrast, female bone was protected against the osteolytic effects of BV6, as no changes in BV/TV or BMD in female mice were observed in the presence of BV6 (Fig. 3 *B*, *C*).

**Figure 4:**
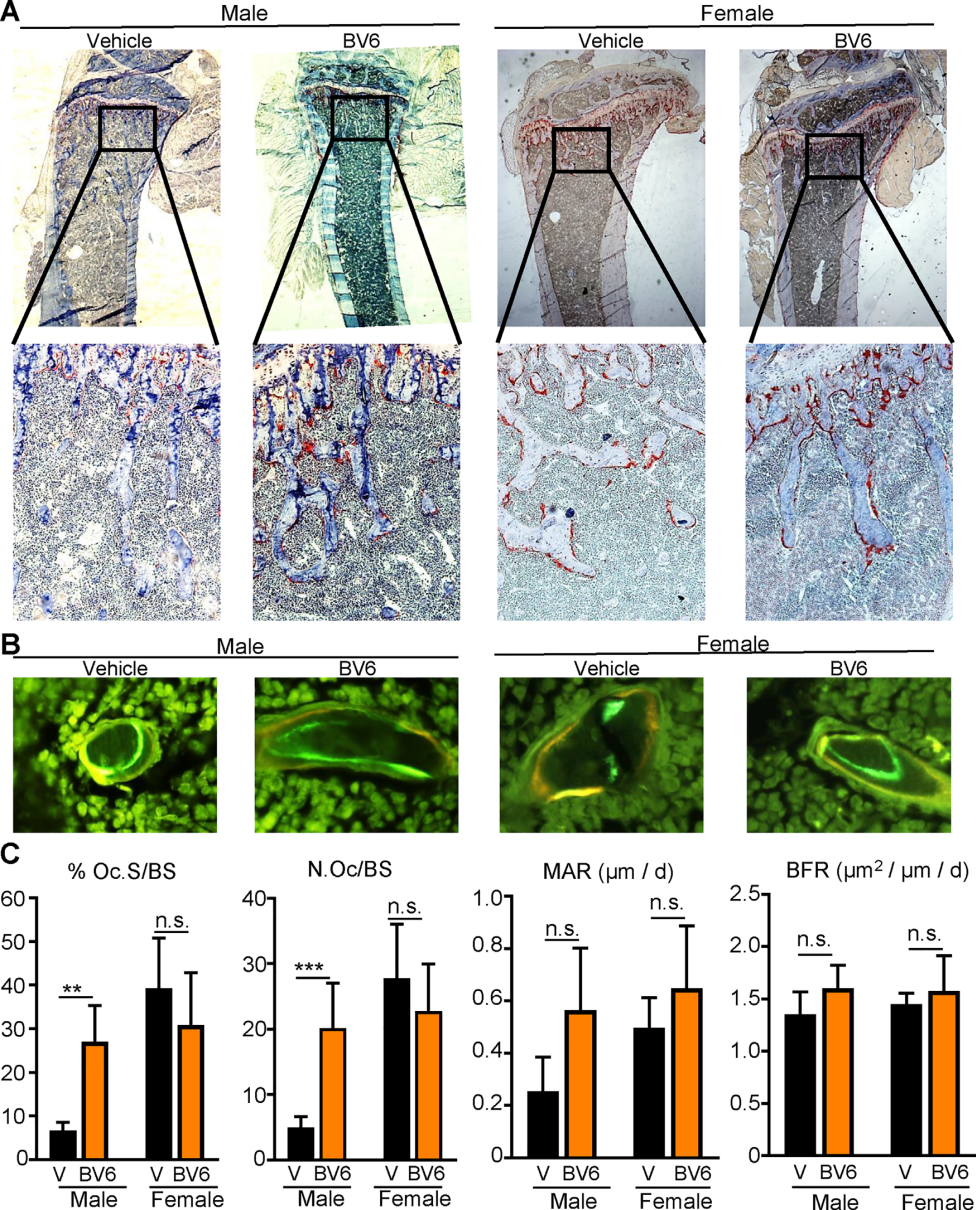
Female osteoclast number and surface do not change with BV6 treatment. (*A*) Tibia of the mice in Fig. 3 were prepared for histomorphometry of TRAP‐stained sections. (*B*) Representative double labeling images of male and female mouse on vehicle or BV6, left to right. (*C*) Histomorphometric analysis of TRAP sections in *A* and bone formation parameters in *B*: Oc.S/BS, N.Oc/BS, MAR, and BFR. Data presented as mean ± SD, **p < 0.01 and ***p < 0.001, two‐way ANOVA. n.s. = not significant; Oc.S/BS = osteoclast surface per bone surface; N.Oc/BS = number of osteoclasts per bone surface; MAR = mineral apposition rate; BFR = bone formation rate.

Histomorphometric analysis showed that male mice receiving BV6 had significantly higher osteoclast surface per bone surface (Fig. 4 *A*, *C*: Oc.S/BS, *p* < 0.01) and number of osteoclasts per bone surface (Fig. 4 *A*, *C*: N.Oc/BS, *p* < 0.001) than vehicle‐treated males, again consistent with our previous results.^8^ However, BV6 had no effects on osteoclast parameters in female mice in comparison with vehicle‐treated controls, correlating with the lack of change in bone mass parameters (Fig. 4 *A*, *C*). In vitro, BV6 increased OC number similarly in both male and female BMMs in concert with RANKL (Supplementary Fig.  2A, B) but had no effects in either sex on osteoblast differentiation from bone marrow stromal cells (Supplementary Fig.  2C, D).

We previously found that BV6 treatment increased mineral apposition rate (MAR) and bone formation rate (BFR) in male mice.^8^ Analysis of nondecalcified unstained sections from this cohort of male and female mice showed the same trend in MAR and BFR with BV6 treatment in males, although neither achieved statistical significance by two‐way ANOVA (Fig. 4 *B*, *C*). Females also showed no effect of BV6 treatment on bone formation parameters (Fig. 4 *B*, *C*). In sum, by both vivaCT and histomorphometry, we were unable to detect any effect of BV6 on bone mass in female mice in vivo.

### OVX does not restore BV6 responsiveness in female mice

**Figure 5:**
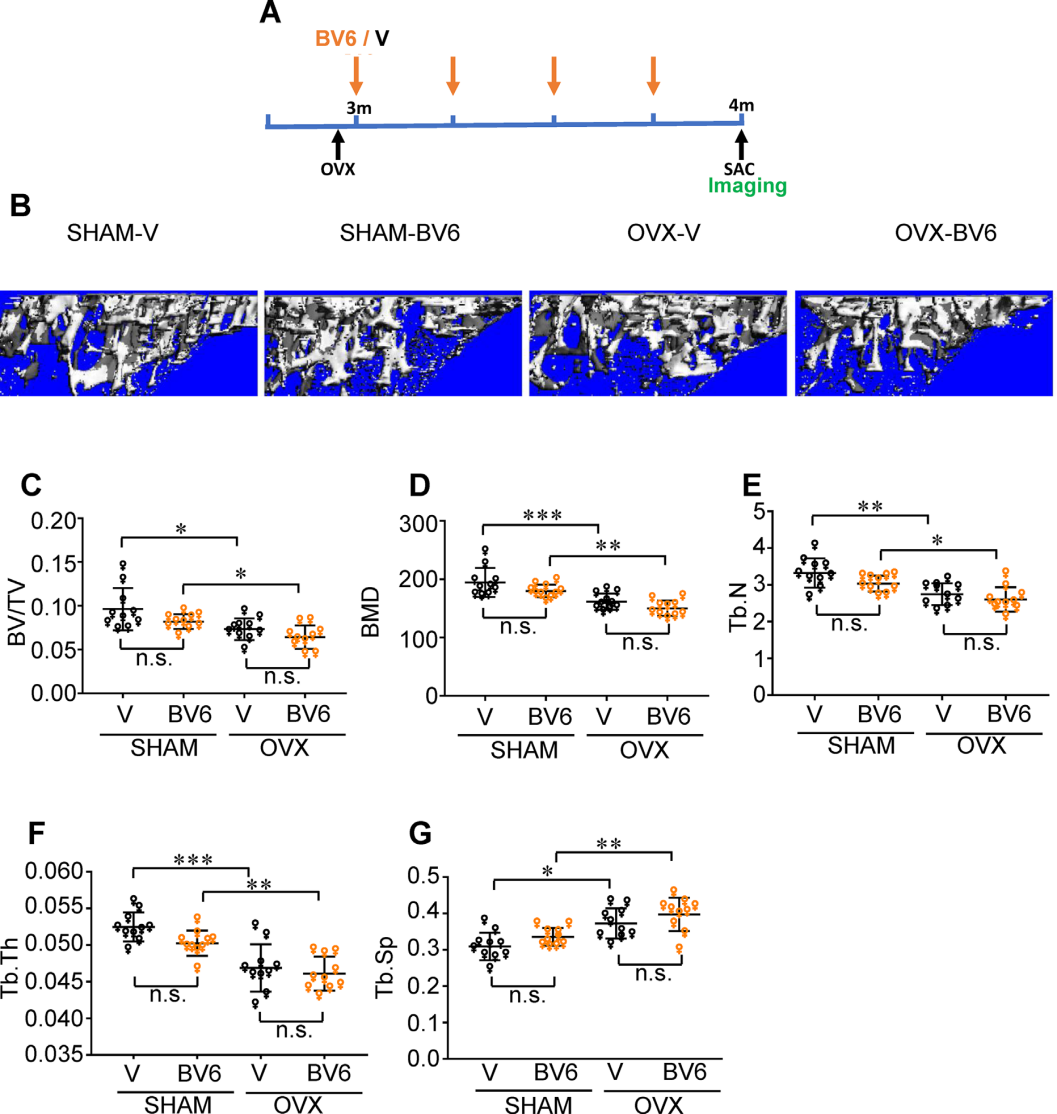
BV6 does not exacerbate bone loss in response to OVX. (*A*) Three‐month‐old WT BALB/c female mice (10/group) were ovariectomized or sham‐operated, then treated with 4 weekly doses of BV6 (orange) (10 mg/kg) or vehicle (black). Mice were euthanized 8 days after the last dose of BV6 and bone parameters were determined. (*B*) μCT three‐dimensional tibia reconstructions of the median mouse in each group. (*C*) BV/TV and (*D*) BMD, (*E*) Tb.N, (*F*) Tb.Th measurements show that OVX mice have lower bone mass than SHAM controls, but BV6 has no additional effect. (*G*) An increase in Tb. Sp was observed in OVX mice on vehicle/BV6 compared to SHAM controls. Data presented as mean ± SD, *p < 0.05, **p < 0.01, and ***p < 0.001, two‐way ANOVA. n.s. = not significant.

Many studies have shown that estrogen impacts NF‐κB signaling in a variety of tissues.^15^ Because we previously showed that BV6 effects on bone are dependent on alternative NF‐κB signaling, we hypothesized that estrogen might interfere with the NF‐κB–activating effects of this drug. To determine if estrogen is responsible for the resistance of females to the effects of BV6 on bone, we performed OVX or sham surgery prior to treatment with BV6 or vehicle over 4 weeks (Fig. 5 *A*, Supplementary Figs.  3 and 4). Confirming our previous observations, none of the bone parameters assessed by μCT were affected in sham groups receiving BV6 compared with sham groups on vehicle (Fig. 5 *B*–*G*). Surprisingly, BV6 also did not change OVX‐induced bone loss in any parameter measured (Fig. 5 *B*–*G*), suggesting that active estrogen signaling is not responsible for the sexual dimorphism of BV6 effects on bone.

## Discussion

We and others have described increases in bone mass in mice with defective components of the alternative NF‐κB pathway, and reciprocal decreases when the pathway is activated.^8^, ^11^, ^12^, ^13^, ^14^, ^26^ However, all of these examined either mixed sex cohorts or only males. With the increased appreciation of the impact of sex differences in pharmacology, and the well‐described interactions between estrogen and NF‐κB signaling, we asked whether there is sexual dimorphism in the alternative NF‐κB phenotypes seen in bone. We found that female mice have a much greater response to loss of either NIK or RelB, both in vivo and in vitro. In contrast, activation of the pathway with the IAP antagonist BV6 decreased bone mass in males, but not females, even following OVX. Thus, there is dramatic sexual dimorphism in these mouse models.

We initially hypothesized that, because estrogen signaling can interfere with NF‐κB activation, female mice might have less pathway activity at baseline than males and therefore would show less response to loss of key components, RelB and NIK. However, we observed the opposite in both RelB‐deficient and NIK‐deficient mice, in which trabecular bone mass was increased greatly in females, and either unchanged or modestly increased in males. Therefore, it is unlikely that antagonism of alternative NF‐κB by estrogen is the primary cause of the observed sexual dimorphism in these genetic models. Interestingly, this difference in severity of phenotype persisted in vitro, where female RelB‐deficient precursors generated fewer OCs than males with RANKL stimulation, and TGFβ failed to stimulate further differentiation. Because male and female cells are exposed to the same mixture of hormones and growth factors present in the fetal calf serum, it is unlikely that the sex difference is the result of direct actions of sex steroids on OC lineage cells.

Based on the more pronounced phenotypes of the genetic models, we predicted that females would respond robustly to pharmacological activation of alternative NF‐κB with BV6. Again, our expectations were controverted by the data. When given to cohorts of male and female mice for 4 weeks beginning at 6 weeks of age, BV6 severely blunted accrual of trabecular bone in males, but had no effect on females. The drug also had no effect on trabecular bone mass in the more mature female sham cohort in the OVX experiment, treated from 12 to 16 weeks of age. Histomorphometry demonstrated a BV6‐induced increase in osteoclasts in males, but not females, in vivo. BV6‐treated males and females did not differ in bone formation rates, suggesting that OCs were indeed responsible for the differential sensitivity between the sexes. However, unlike the genetic models, this difference was not reflected in WT mice in vitro, where male and female OCs were equally stimulated by the drug. It is possible that the overall effect of BV6 on bone resorption in vivo is modulated by other factors not recapitulated in culture. It is also possible that the drug exposure regimen in vitro (added to culture daily for 2 hours) has different effects than that used in vivo (once per week, half‐life ∼2 hours). Alternate day treatment in vitro is similar to daily treatment, and a single stimulus at the beginning of culture has minimal effects on either sex (not shown). Thus, we favor the likelihood that in vivo factors are responsible for the sexual dimorphism in drug response.

The significant impact of loss of alternative NF‐κB on basal bone mass in the genetic models using estrogen‐sufficient females suggests that estrogen does not prevent activation of this pathway at physiological levels. However, it seemed plausible that estrogen could counteract BV6‐induced alternative NF‐κB, rendering female mice insensitive to the drug's osteolytic activity. We therefore decided to compare the response of intact and ovariectomized females to BV6, expecting reduced estrogen levels to sensitize mice to the drug. Although OVX caused the expected loss in bone mass, BV6 did not cause any further osteolysis. Therefore, the resistance of females to BV6 is not likely related to interference by estrogen. In sum, none of our data support a direct role for estrogen in the observed sexual dimorphisms related to alternative NF‐κB in bone.

Another potential explanation for our findings is that, under basal conditions, the NIK pathway is strongly active in females and minimally active in males, such that its loss impacts females to a much greater extent. BV6 promotes NIK signaling, but the more pronounced effect in males might occur because their baseline activity of the pathway is lower than females. Because of a lack of pathway‐specific reporters and downstream targets, it is currently very difficult to assess alternative NF‐κB activation in vivo. Therefore, determining the validity of this hypothesis will require significant future experimentation. Furthermore, at this point we cannot speculate on a specific mechanism by which differential utilization of NIK is regulated by sex, although epigenetic modulation of expression of key components is the most likely explanation because there are known sex differences in other organs.^27^, ^28^, ^29^

Regardless of the molecular basis of the sexual dimorphism, the difference in response of the bone to IAP antagonist treatment may have clinical implications. This class of drugs will likely be used for solid tumors in combination with cytotoxic agents that are known to cause bone loss,^30^, ^31^ and although osteoporotic fractures may not be a primary concern in cancer patients, they can have a very significant impact on quality of life. Because we found no osteoporotic effect in female mice, even with OVX, there should be less concern for therapy‐related effects of IAP antagonists in women, who have a higher incidence of osteoporosis than men.^32^ Our previous study demonstrated an increase in the incidence and severity of bone metastases in male mice inoculated with BV6‐resistant tumors, which we attributed to the osteoclastogenic effects of the drug.^8^ Based on our current study, we would expect a smaller pro‐metastatic effect in females. Therefore, in women with breast cancer, IAP antagonists may have a minimal risk of increasing either bone metastasis or osteoporosis, both a significant concern in this patient population. However, in lung cancer, which has a high rate of bone metastasis and a higher prevalence in men, IAP antagonists might carry a significant risk of adverse bone‐related effects in men. Further work is needed to determine whether the sex difference in IAP antagonist effects on bone occur in the context of bone metastasis.

## Disclosures

All authors state that they have no conflicts of interest

## Supporting information

Supporting Figures S1.

## References

[1] Novack DV. Role of NF‐κB in the skeleton. Cell Res. 2011;21(1):169–82. 21079651 10.1038/cr.2010.159 PMC3193402

[2] Sun SC. The non‐canonical NF‐κB pathway in immunity and inflammation. Nat Rev Immunol. 2017;17 (9):545–58. 28580957 10.1038/nri.2017.52 PMC5753586

[3] Gyrd‐Hansen M , Meier P. IAPs: from caspase inhibitors to modulators of NF‐κB, inflammation and cancer. Nat Rev Cancer. 2010;10 (8):561–74. 20651737 10.1038/nrc2889

[4] Li L , Thomas RM , Suzuki H , De Brabander JK , Wang X , Harran PG. A small molecule Smac mimic potentiates TRAIL‐ and TNFα‐mediated cell death. Science. 2004;305 (5689):1471–4. 15353805 10.1126/science.1098231

[5] Finlay D , Teriete P , Vamos M , Cosford ND , Vuori K. Inducing death in tumor cells: roles of the inhibitor of apoptosis proteins. F1000Res. 2017;6(F1000 Faculty Rev):587 DOI:10.12688/f1000research.10625.1. 28529715 PMC5414821

[6] Suva LJ , Washam C , Nicholas RW , Griffin RJ. Bone metastasis: mechanisms and therapeutic opportunities. Nat Rev Endocrinol. 2011;7 (4):208–18. 21200394 10.1038/nrendo.2010.227 PMC3134309

[7] Weilbaecher KN , Guise TA , McCauley LK. Cancer to bone: a fatal attraction. Nat Rev Cancer. 2011;11 (6):411–25. 21593787 10.1038/nrc3055 PMC3666847

[8] Yang C , Davis JL , Zeng R , et al. Antagonism of inhibitor of apoptosis proteins increases bone metastasis via unexpected osteoclast activation. Cancer Discov. 2013;3 (2):212–23. 23269702 10.1158/2159-8290.CD-12-0271 PMC3570610

[9] Seeman E. Clinical review 137: Sexual dimorphism in skeletal size, density, and strength. J Clin Endocrinol Metab. 2001;86 (10):4576–84. 11600506 10.1210/jcem.86.10.7960

[10] Laurent M , Antonio L , Sinnesael M , et al. Androgens and estrogens in skeletal sexual dimorphism. Asian J Androl. 2014;16 (2):213. 24385015 10.4103/1008-682X.122356 PMC3955330

[11] Yao Z , Li Y , Yin X , Dong Y , Xing L , Boyce BF. NF‐κB RelB negatively regulates osteoblast differentiation and bone formation. J Bone Miner Res. 2014;29 (4):866–77. 24115294 10.1002/jbmr.2108 PMC3961566

[12] Soysa NS , Alles N , Weih D , et al. The pivotal role of the alternative NF‐κB pathway in maintenance of basal bone homeostasis and osteoclastogenesis. J Bone Miner Res. 2010;25 (4):809–18. 19839765 10.1359/jbmr.091030

[13] Maruyama T , Fukushima H , Nakao K , et al. Processing of the NF‐κ B2 precursor p100 to p52 is critical for RANKL‐induced osteoclast differentiation. J Bone Miner Res. 2010;25 (5):1058–67. 19874202 10.1359/jbmr.091032

[14] Vaira S , Johnson T , Hirbe AC , et al. RelB is the NF‐κB subunit downstream of NIK responsible for osteoclast differentiation. Proc Natl Acad Sci U S A. 2008;105 (10):3897–902. 18322009 10.1073/pnas.0708576105 PMC2268780

[15] Kalaitzidis D , Gilmore TD. Transcription factor cross‐talk: the estrogen receptor and NF‐κB. Trends Endocrinol Metab. 2005;16 (2):46–52. 15734144 10.1016/j.tem.2005.01.004

[16] Sas L , Lardon F , Vermeulen PB , et al. The interaction between ER and NFκB in resistance to endocrine therapy. Breast Cancer Res. 2012;14 (4):212. 22963717 10.1186/bcr3196 PMC3680926

[17] Wang X , Belguise K , Kersual N , et al. Oestrogen signalling inhibits invasive phenotype by repressing RelB and its target BCL2. Nat Cell Biol. 2007;9(4):470–8. 17369819 10.1038/ncb1559 PMC2394707

[18] Nakamura T , Imai Y , Matsumoto T , et al. Estrogen prevents bone loss via estrogen receptor α and induction of Fas ligand in osteoclasts. Cell. 2007;130 (5):811–23. 17803905 10.1016/j.cell.2007.07.025

[19] Takeshita S , Kaji K , Kudo A. Identification and characterization of the new osteoclast progenitor with macrophage phenotypes being able to differentiate into mature osteoclasts. J Bone Miner Res. 2000;15 (8):1477–88. 10934646 10.1359/jbmr.2000.15.8.1477

[20] McHugh KP , Hodivala‐Dilke K , Zheng MH , et al. Mice lacking β3 integrins are osteosclerotic because of dysfunctional osteoclasts. J Clin Invest. 2000;105 (4):433–40. 10683372 10.1172/JCI8905 PMC289172

[21] Yin L , Wu L , Wesche H , et al. Defective lymphotoxin‐β receptor‐induced NF‐κaB transcriptional activity in NIK‐deficient mice. Science. 2001;291 (5511):2162–5. 11251123 10.1126/science.1058453

[22] Weih F , Carrasco D , Durham SK , et al. Multiorgan inflammation and hematopoietic abnormalities in mice with a targeted disruption of RelB, a member of the NF‐κ B/Rel family. Cell. 1995;80 (2):331–40. 7834753 10.1016/0092-8674(95)90416-6

[23] Itonaga I , Sabokbar A , Sun SG , et al. Transforming growth factor‐β induces osteoclast formation in the absence of RANKL. Bone. 2004;34 (1):57–64. 14751563 10.1016/j.bone.2003.08.008

[24] Quinn JM , Whitty GA , Byrne RJ , Gillespie MT , Hamilton JA. The generation of highly enriched osteoclast‐lineage cell populations. Bone. 2002;30 (1):164–70. 11792580 10.1016/s8756-3282(01)00654-8

[25] Park JH , Lee NK , Lee SY. Current understanding of RANK signaling in osteoclast differentiation and maturation. Mol Cells. 2017;40 (10):706. 29047262 10.14348/molcells.2017.0225 PMC5682248

[26] Yang C , McCoy K , Davis JL , et al. NIK stabilization in osteoclasts results in osteoporosis and enhanced inflammatory osteolysis. PLoS One. 2010;5 (11):e15383. 21151480 10.1371/journal.pone.0015383 PMC2975662

[27] Khulan B , Cooper WN , Skinner BM , et al. Periconceptional maternal micronutrient supplementation is associated with widespread gender related changes in the epigenome: a study of a unique resource in the Gambia. Hum Mol Genet. 2012;21 (9):2086–101. 22307237 10.1093/hmg/dds026

[28] Qureshi IA , Mehler MF . Genetic and epigenetic underpinnings of sex differences in the brain and in neurological and psychiatric disease susceptibility. Prog Brain Res. 2010;186:77–95. 21094887 10.1016/B978-0-444-53630-3.00006-3 PMC4465286

[29] Gabory A , Attig L , Junien C. Sexual dimorphism in environmental epigenetic programming. Mol Cell Endocrinol. 2009;304(1–2):8–18. 19433243 10.1016/j.mce.2009.02.015

[30] Yang C , Novack DV. Anti‐cancer IAP antagonists promote bone metastasis: a cautionary tale. J Bone Miner Metab. 2013;31 (5):496–506. 23740289 10.1007/s00774-013-0479-0 PMC3962044

[31] Mehrotra S , Languino LR , Raskett CM , Mercurio AM , Dohi T , Altieri DC. IAP regulation of metastasis. Cancer Cell. 2010;17 (1):53–64. 20129247 10.1016/j.ccr.2009.11.021 PMC2818597

[32] Alswat KA . Gender disparities in osteoporosis. J Clin Med Res. 2017;9 (5):382. 28392857 10.14740/jocmr2970w PMC5380170

