# Supplementary material for: Manipulation of the Alternative NF‐κB Pathway in Mice Has Sexually Dimorphic Effects on Bone
Source: JBMR Plus. 2018 Aug 23;3(1):14–22. doi: 10.1002/jbm4.10066 (PMC6339559; doi:10.1002/jbm4.10066)
Supplement: Supplementary file 1 — Supporting Figures S1. [file JBM4-3-14-s001.pdf]

# Manipulation of the alternative NF-κB pathway in mice has sexually dimorphic effects on bone

Allahdad Zarei, Chang Yang, Jesse Gibbs, Jennifer L. Davis, Anna Ballard, Rong Zeng, Linda Cox, Deborah J. Veis.

## Supplementary 1

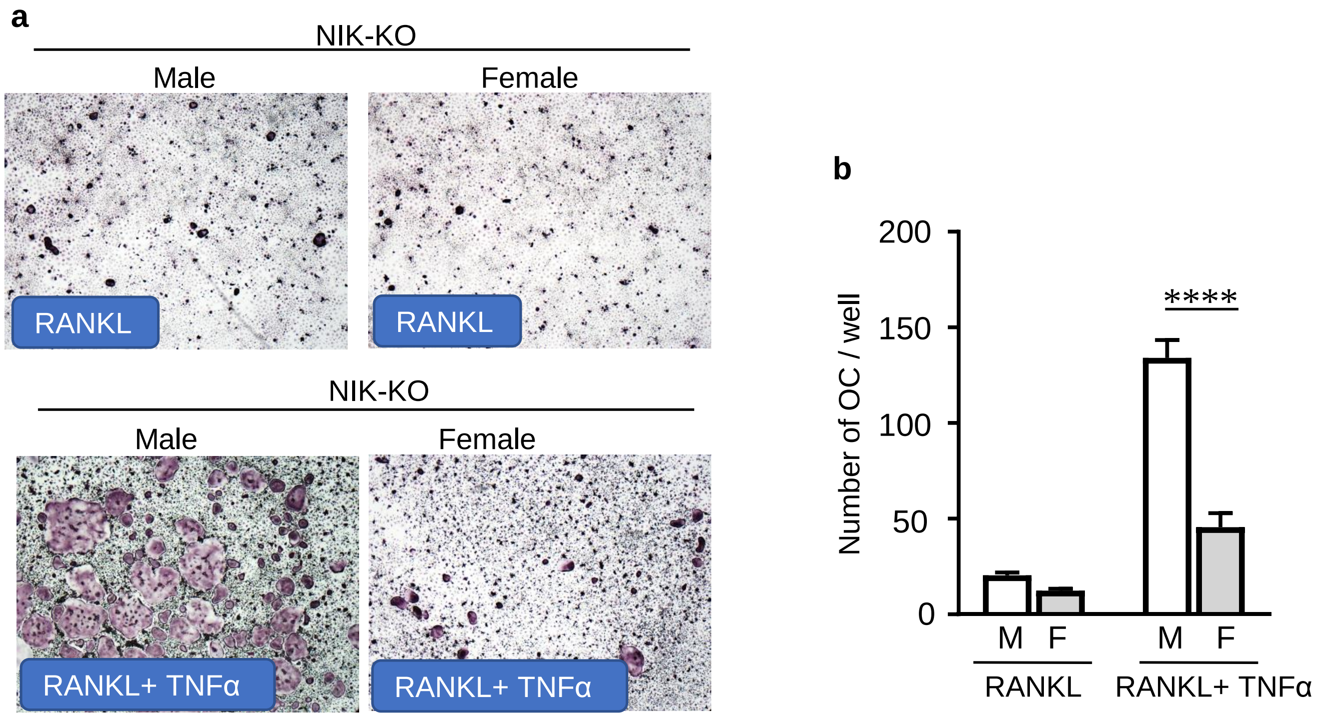


**Osteoclastogenesis is highly suppressed in female NIK-KO mice in vitro.** (a) RANKL-induced (top row) osteoclast formation from male and female NIK-KO mice demonstrating suppression of osteoclastogenesis in both sexes. Addition of TNFα with RANKL (bottom row) generates Ocs in male BMMs but not females. (b) TRAP positive cells with ≥3 nuclei in (a) were counted in each well and plotted, white presence of RANKL, grey RANKL + TNFα. Data presented as mean ±S.D, Two-Way ANOVA, **** $p<0.0001$.

# Manipulation of the alternative NF-κB pathway in mice has sexually dimorphic effects on bone

Allahdad Zarei, Chang Yang, Jesse Gibbs, Jennifer L. Davis, Anna Ballard, Rong Zeng, Linda Cox, Deborah J. Veis.

## Supplementary 2

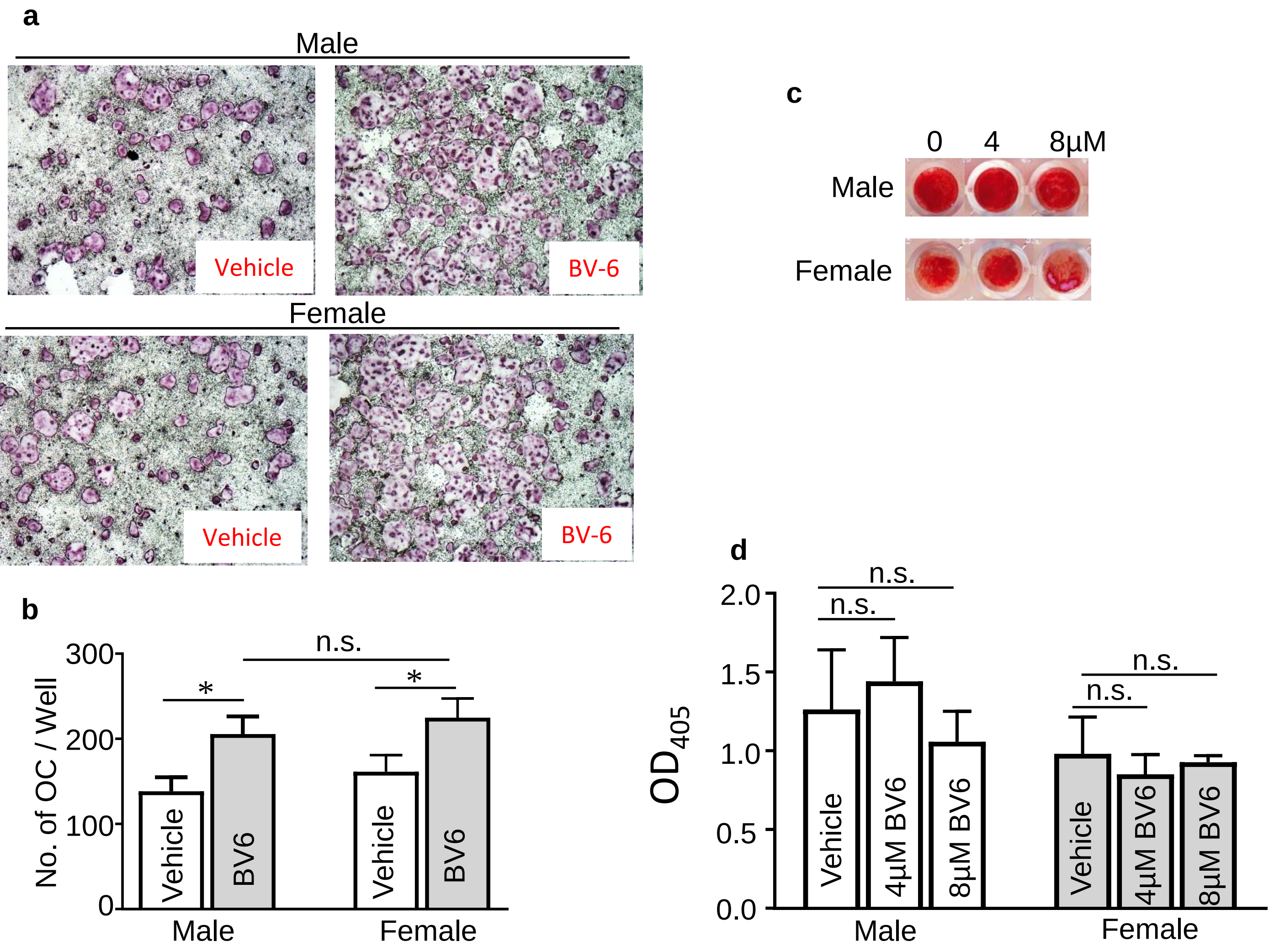


**BV6 enhances OC formation but not osteoblast differentiation, *in vitro* in both male and female WT mice.** (a) BMMs from WT C57BL/6 male and female mice were cultured with 5ng/ml RANKL ± 8µM BV6 or vehicle. (b) TRAP positive cells with ≥3 nuclei were counted in each well and plotted. (c) Tibias and femurs were dissected from 3 WT male and female mice, spun at 10,000 RPM for two minutes in sterile eppendorf. Bone marrows were filtered with 0.7 micron filter and cultured in αMEM containing 10%FBS, 1% Penicillin-Streptomycin and L-glutamine in 150mm culture plates for 10 days. Cells were re-plated at $4x10^4$ per well in 96-well plate format in quadruplicate in osteogenic media (αMEM 10%FBS, 50µg/ml L-ascorbic acid and 10mM β-glycerol 2-phosphate disodium salt) for 11 days. Upon completion of the assa,y media was removed, cells were fixed with 70% ethanol for 1 hour, washed with distilled water and stained with 1% Alizarin red dye solution. After staining cells were washed with deionized water, air dried, and photographed. One representative mouse shown for each condition. (d). Wells from c were extracted by addition of 100µl 10% acetic acid over orbital shaker and alizarin levels measured colorimetrically at 405 nm Data presented as mean ±S.D from 3 male and 3 female mice, Two-Way ANOVA, * p<0.05.

# Manipulation of the alternative NF-κB pathway in mice has sexually dimorphic effects on bone

Allahdad Zarei, Chang Yang, Jesse Gibbs, Jennifer L. Davis, Anna Ballard, Rong Zeng, Linda Cox, Deborah J. Veis.

## Supplementary 3

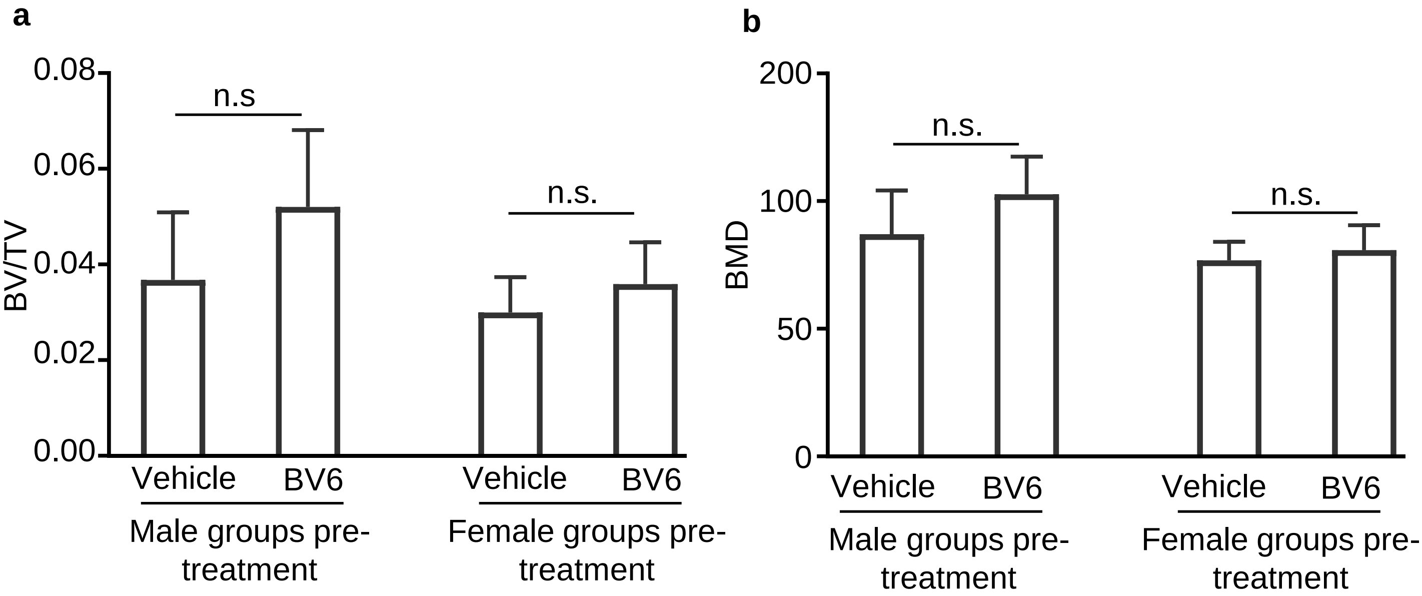


**Vehicle or BV6 pre-treated male and female groups had similar bone phenotype.** VivaCT analysis of (a) BV/TV and (b) BMD in WT BALB/c mice, age 6 weeks, male and female (n=7-8 /group) prior to receiving either vehicle or BV6, Mean ± S.D, two-Way ANOVA, n.s. not significant.

# Manipulation of the alternative NF-κB pathway in mice has sexually dimorphic effects on bone

Allahdad Zarei, Chang Yang, Jesse Gibbs, Jennifer L. Davis, Anna Ballard, Rong Zeng, Linda Cox, Deborah J. Veis.

## Supplementary 4

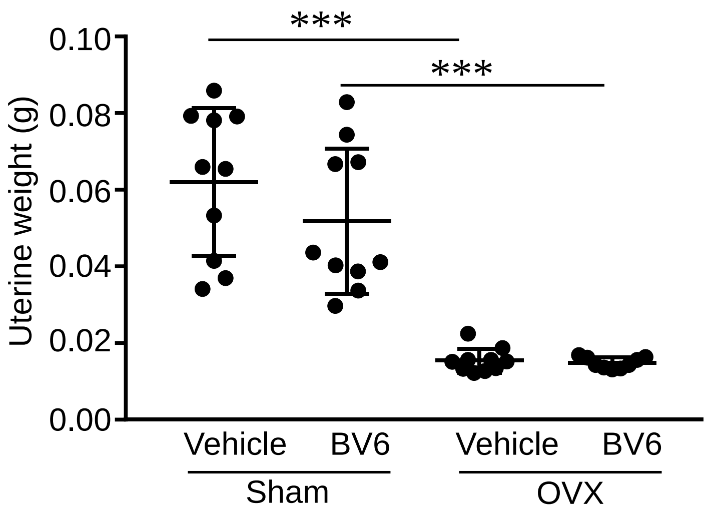


**Uterine weight decreases in ovariectomized mice.** Significant decrease was observed in uterine weight of ovariectomized mice treated with BV6 or vehicle compared to sham groups. There was no difference between vehicle and BV6 within treatment groups. Mean ± S.D, n=9-10 /group, two-Way ANOVA, *** p<0.001.
